# Supplementary material for: Coping with intimate partner violence and the COVID-19 lockdown: The perspectives of service professionals in Spain
Source: PLoS One. 2021 Oct 21;16(10):e0258865. doi: 10.1371/journal.pone.0258865 (PMC8530357; doi:10.1371/journal.pone.0258865)
Supplement: S1 Table — (DOCX) [file pone.0258865.s001.docx]

S1 Table. COREQ CHECKLIST. Consolidated criteria for reporting qualitative research

| No. Item | Guide questions/description | Reported |
| --- | --- | --- |
| Domain 1: Research team an reﬂexivity | | |
| 1. Inter viewer/facilitator | Which author/s conducted the  interview? | *The interviews were conducted by three interviewers from the research team with training and experience in qualitative research methodology* |
| 2. Credentials | What were the researcher’s credentials? |  |
| 3. Occupation | What was their occupation at the time of the study? |  |
| 4. Gender | Was the researcher male or female? | *The interviews were conducted by three female researchers* |
| 5. Experience and training | What experience or training did the researcher have? | *Interviewers have sufficient training and experience in qualitative research methodology* |
| 6. Relationship with participants established | Was a relationship established prior to study commencement? | *No* |
| 7. Participant knowledge  of the interviewer | What did the participants know about  the researcher? | *The study participants were informed at the time of contact and at the beginning of the interviews, about the project, the research leader and the university responsible of it, their scope and study objectives .* |
| 8. Interviewer  characteristics | What characteristics were reported  about the inter viewer/facilitator? |  |
| Domain 2: study design | | |
| 9. Methodological  orientation and Theory | What methodological orientation was  stated to underpin the study? | *Content analysis* |
| 10. Sampling | How were participants selected? | *Participants were identified, selected and recruited by an initial online prospection of potential participants, dissemination of the study online and following a snowball strategy* |
| 11. Method of approach | How were participants approached? | *An initial email was sent to each IPV-response service with details of the University leader, research team and study objectives to encourage the participation in the research* |
| 12. Sample size | How many participants were in the study? | *47 professionals: 44 women and 3 men, from 40 different entities were interviewed* |
| 13. Non-­‐participation | How many people refused to participate or dropped out? Reasons? | *This information was not recorded.* |
| 14. Setting of data collection | Where was the data collected? | *Interviews were carried out by telephone and video call* |
| 15. Presence of non-­‐  participants | Was anyone else present besides the  participants and researchers? | *No* |
| 16. Description of sample | What are the important characteristics of the sample? | *The heterogeneity of the sample seeking the representativeness of the services in charge of IPV against women response: 1)* *type of resource, 2) activity area, 3) area of care,*  *4) professional profile, 5) geographic distribution, and 6) representation of specialized resources for groups in situations of vulnerability* |
| 17. Interview guide | Were questions, prompts, guides provided by the authors? | *Interview guide was piloted and adapted in the first phases of the field work in joint work sessions with different members of the research team* |
| 18. Repeat interviews | Were repeat interviews carried out? | *No* |
| 19. Audio/visual recording | Did the research use audio or visual recording to collect the data? | *Audio recording was used to collect data from telephone and video call interviews* |
| 20. Field notes | Were ﬁeld notes made during and/or after the interview? | *Field notes were recorded at the end of each interview, making difference: a) information from the context of the interview and communication, b) principle ideas emerged from the discourse. Those were used by the analysts to approach the interviews.* |
| 21. Duration | What was the duration of the interviews? | *The duration of the interviews was from 50 to 70 minutes* |
| 22. Data saturation | Was data saturation discussed? | *The research team considered that data saturation was achieved when latest interviews did not generate new additional information*. |
| 23. Transcripts returned | Were transcripts returned to  participants for comment and/or correction? | *No* |
| Domain 3: analysis and ﬁndings | | |
| 24. Number of data coders | How many data coders coded the data? | *Three analyst from the research team* |
| 25. Description of the  coding tree | Did authors provide a description of the  coding tree? | *Yes and we provide the coding tree as a supplementary annex.* |
| 26. Derivation of themes | Were themes identiﬁed in advance or derived from the data? | *Categories and codes used for analyzing the transcriptions was based on the study objectives and a preliminary analysis of information from the interviews. New codes that emerged were added during the process of analysis* |
| 27. Software | What software, if applicable, was used to manage the data? | *Atlas.ti (version 9)* |
| 28. Participant checking | Did participants provide feedback on the ﬁndings? | *Because of the overload work of the professionals from IPV-response services we decide not to recontact them, so feedback was not collected.* |
| 29. Quotations presented | Were participant quotations presented to illustrate the themes/ﬁndings? Was  each quotation identiﬁed? | *Identified quotations are added in the manuscript in results section* |
| 30. Data and ﬁndings consistent | Was there consistency between the data presented and the ﬁndings? | *Yes* |
| 31. Clarity of major themes | Were major themes clearly presented in the ﬁndings? | Yes |
| 32. Clarity of minor  themes | Is there a description of diverse cases or discussion of minor themes? | Yes |
