# Supplementary material for: Coping with intimate partner violence and the COVID-19 lockdown: The perspectives of service professionals in Spain
Source: PLoS One. 2021 Oct 21;16(10):e0258865. doi: 10.1371/journal.pone.0258865 (PMC8530357; doi:10.1371/journal.pone.0258865)
Supplement: S2 Table — (DOCX) [file pone.0258865.s002.docx]

S2 Table. Interview guide questions

| Main domain | Questions |
| --- | --- |
| 1. Introduction questions | Your work entity objectives and scope  Your previous career  ¿How was your work in relation to intimate partner violence attention in the months before the COVID-19 lockdown? |
| 1. Context and needs of the women | 1.1 What implications do you remember that the declaration of the State of Alarm had in relation to your work related to attention to intimate partner violence?  1.2 Did you observe any change in situations of violence during the state of alarm?  1.3 Which women were most vulnerable in the confinement situation?  1.4 What were the main demands of women in situations of violence during confinement? |
| 1. Strategies adopted for the services and working conditions | 2.1 What response was given by your entity to the attention to intimate partner violence during lockdown (activated resources, etc.)?  2.4 How was the coordination with other sectors/institutions usually involved in the care of women in situations of violence that is provided from your institution (e.g. shelters, municipal social services...)?  2.3 Do you think that the response of professionals to a demand or suspicion of intimate partner violence during confinement has been different than it would have been before in a regular situation? |
| 1. Lived work experience (obstacles, positive aspects and proposals for improvement) | 3.1. What positive elements do you identify in the response given by your entity to the attention to intimate partner violence throughout these months, since the beginning of the pandemic?  3.2. What were the main obstacles/limitations to develop your work throughout these months since the beginning of the pandemic?  3.3. What would it have taken to provide a better response?  3.4. Based on your experience, what do you think may be some key strategies for preventing intimate partner violence during confinement? Specifically, for the prevention of new cases, aggravation, and recidivism.  3.5. What recommendations would you make to address intimate partner violence cases in the coming months?  3.6. What would you be left with as the most positive experience of all that has happened since the beginning of the pandemic to the present day? |
