# Supplementary material for: Coping with intimate partner violence and the COVID-19 lockdown: The perspectives of service professionals in Spain
Source: PLoS One. 2021 Oct 21;16(10):e0258865. doi: 10.1371/journal.pone.0258865 (PMC8530357; doi:10.1371/journal.pone.0258865)
Supplement: S3 Table — (DOCX) [file pone.0258865.s003.docx]

S3 Table. Coding tree

Intimate Partner Violence and the COVID-19 lockdown: The Perspectives of Service Professionals in Spain

| **BLOCK** | **CATEGORIES** | **CODES** | **DESCRIPTION** |
| --- | --- | --- | --- |
| **CONFINEMENT (BEGINNING OF THE PANDEMIC)** | **WORK EXPERIENCE** | **Response_confinement_general** | Given response from the services to people who come for any reason during confinement period (presence or absence of changes/adaptation). Continuation or interruption of the response to people who were already users of the resource (confinement and prepandemic period). |
|  |  | **Response_confinement_violence** | Given response from the services to women victims of IPV during confinement period (presence or absence of changes/adaptation). Continuation or interruption of the response to people who were already users of the resource (confinement and prepandemic period). |
|  |  | **Internal organization_ confinement** | Internal organization of the services to deliver a response during confinement period (teams, functions, means and working conditions). Changes and / or continuities from the prior periods (confinement and prepandemic period). |
|  |  | **Coordination_services_ confinement** | Presence or absence of coordination between services during confinement period. How this coordination is articulated (communication channels, teams and means). Changes and / or continuities from the prior periods (confinement and prepandemic period). |
|  |  | **Working conditions_ confinement** | Impact of the confinement period on professionals in psycho-emotional terms and repercussion on the quality of work, their ability to perform it and the response from the resource. It includes the impact of the workers' personal situations, particularly their role as caregivers (of children, people with specific needs, etc.). |
|  | **CONTEXT OF CARE** | **Characteristic _violence_ confinement** | Description of the situations of violence experienced during the confinement period (frequency, incidence, types, etc.). |
|  |  | **Context_ confinement** | Contexts of the situation of women during the confinement period beyond those directly related to violence (socioeconomic, work, family, personal, etc.). |
|  |  | **Use_service_ confinement** | Existence or not of user appointment at the services during the confinement period. Characteristics, quantity and frequency of the consultations. Existence or not of new users of the services during the pandemic and / or continuity of the previous ones. Belonging or not of women to specific groups of vulnerability. |
|  | **DEMANDS** | **Demands_victims_ confinement** | Demands arising in relation to the impact of the confinement (on their situation of violence or on other areas). Types of demands. Who transfers the demands. Existence or not of changes with respect to the previous period (confinement and prepandemic). |
|  |  | **Needs_children_ confinement** | Needs of the children arising in relation to the impact of the confinement period (on the situation of violence or on other areas). Types of needs. Existence or not of changes with respect to the previous period (confinement and prepandemic). |
|  | | | |
| **DE-ESCALATION PERIOD** | **WORK EXPERIENCE** | **Response _descaled_general** | Given response from the services to people who come for any reason during descaled period (presence or absence of changes/adaptation). Continuation or interruption of the response to people who were already users of the resource (confinement and prepandemic period). |
|  |  | **Response _descaled_violence** | Given response from the services to women victims of IPV during descaled period (presence or absence of changes/adaptation). Continuation or interruption of the response to people who were already users of the resource (confinement and prepandemic period). |
|  |  | **Internal organization _descaled** | Internal organization of the services to deliver a response during descaled period (teams, functions, means and working conditions). Changes and / or continuities from the prior periods (confinement and prepandemic period). |
|  |  | **Coordination_services _descaled** | Presence or absence of coordination between services during descaled period. How this coordination is articulated (communication channels, teams and means). Changes and / or continuities from the prior periods (confinement and prepandemic period). |
|  |  | **Working conditions_descaled** | Impact of the descaled period on professionals in psycho-emotional terms and repercussion on the quality of work, their ability to perform it and the response from the resource. It includes the impact of the workers' personal situations, particularly their role as caregivers (of children, people with specific needs, etc.). |
|  | **CONTEXT OF CARE** | **Characteristic _violence _descaled** | Description of the situations of violence experienced during the descaled period (frequency, incidence, types, etc.). |
|  |  | **Context_descaled** | Contexts of the situation of women during the descaled period beyond those directly related to violence (socioeconomic, work, family, personal, etc.). |
|  |  | **Service´s use _descaled** | Existence or not of user appointment at the services during the descaled period. Characteristics, quantity and frequency of the consultations. Existence or not of new users of the services during the pandemic and / or continuity of the previous ones. Belonging or not of women to specific groups of vulnerability. |
|  | **DEMANDS** | **Demands_victims_descaled** | Demands arising in relation to the impact of the descaled period (on their situation of violence or on other areas). Types of demands. Who transfers the demands. Existence or not of changes with respect to the previous period (confinement and prepandemic). |
|  |  | **Needs_children_descaled** | Needs of the children arising in relation to the impact of the descaled period (on the situation of violence or on other areas). Types of needs. Existence or not of changes with respect to the previous period (confinement and prepandemic). |
|  | | | |
| **CURRENTLY (PERIOD AFTER DE-ESCALATION)** | **WORK EXPERIENCE** | **Response_current_general** | Given response from the services to people who come for any reason during transitional months and the current moment (presence or absence of changes/adaptation). Continuation or interruption of the previous work (previous phases and prepandemic period). |
|  |  | **Response_current_violence** | Given response from the services to women victims of IPV during transitional months and the current moment (presence or absence of changes/adaptation). Continuation or interruption of the previous work (previous phases and prepandemic period). |
|  |  | **Internal organization _current** | Internal organization of the services to deliver a response during during transitional months and the current moment (teams, functions, means and working conditions). Changes and / or continuities from the prior periods (previous phases and prepandemic period). |
|  |  | **Coordination_services_ current** | Presence or absence of coordination between services during transitional months and the current moment. How this coordination is articulated (communication channels, teams and means). Changes and / or continuities from the prior periods (previous phases and prepandemic period). |
|  |  | **Working conditions_current** | Impact of the transitional months and the current moment on professionals in psycho-emotional terms and repercussion on the quality of work, their ability to perform it and the response from the resource. It includes the impact of the workers' personal situations, particularly their role as caregivers (of children, people with specific needs, etc.). |
|  | **CONTEXT OF CARE** | **Characteristic _violence_current** | Description of the situations of violence experienced during the transitional months and the current moment (frequency, incidence, types, etc.). |
|  |  | **Context_current** | Contexts of the situation of women during the transitional months and the current moment, beyond those directly related to violence (socioeconomic, work, family, personal, etc.). |
|  |  | **Service´s use_current** | Existence or not of user appointment at the services during the transitional months and the current moment. Characteristics, quantity and frequency of the consultations. Existence or not of new users of the services during the pandemic and / or continuity of the previous ones. Belonging or not of women to specific groups of vulnerability. |
|  | **DEMANDS** | **Demands_victims_current** | Demands arising in relation to the impact of the transitional months and the current moment (on their situation of violence or on other areas). Types of demands. Who transfers the demands. Existence or not of changes with respect to the previous period (previous phases and prepandemic). |
|  |  | **Needs_children_current** | Needs of the children arising in relation to the impact of the transitional months and the current moment (on the situation of violence or on other areas). Types of needs. Existence or not of changes with respect to the previous period (previous phases and prepandemic). |
|  | | | |
| **CROSS-SECTIONAL** | **WORK EXPERIENCE** | **Response_transversal_general** | Given response from the services to people who come for any reason during the pandemic (presence or absence of changes/adaptation). Continuation or interruption of the care to women who were already users of the service before the beginning of the pandemic. |
|  |  | **Response_transversal_violence** | Given response from the services to women victims of IPV during the pandemic (presence or absence of changes/adaptation). Continuation or interruption of the care to women who were already users of the service before the beginning of the pandemic. |
|  |  | **Internal organization_transversal** | Internal organization of the services to deliver a response during the pandemic period (teams, functions, means and working conditions). Changes and / or continuities from the prior period of the pandemic. |
|  |  | **Coordination_services_transversal** | Presence or absence of coordination between services during the pandemic. How this coordination is articulated (communication channels, teams and means). Changes and / or continuities from the prior period of the pandemic. |
|  |  | **Working conditions_transversal** | Impact of the pandemic on professionals in psycho-emotional terms and repercussion on the quality of work, their ability to perform it and the response from the resource. It includes the impact of the workers' personal situations, particularly their role as caregivers (of children, people with specific needs, etc.). |
|  | **CONTEXT OF CARE** | **Characteristic _violence _transversal** | Description of the situations of violence experienced during the pandemic (frequency, incidence, types, etc.) |
|  |  | **Context_transversal** | Contexts of the situation of women during the pandemic beyond those directly related to violence (socioeconomic, work, family, personal, etc.). |
|  |  | **Service´s use _transversal** | Existence or not of user appointment at the services during the pandemic. Characteristics, quantity and frequency of the consultations. Existence or not of new users of the services during the pandemic and / or continuity of the previous ones. Belonging or not of women to specific groups of vulnerability. |
|  | **DEMANDS** | **Demands_victims_transversal** | Demands of women arising in relation to the impact of the pandemic (on their situation of violence or on other areas). Types of demands. Who transfers the demands. Existence or not of changes compared to the period prior to the pandemic. |
|  |  | **Needs_children_transversal** | Needs of the children arising in relation to the impact of the pandemic (on the situation of violence or on other areas). Types of needs. Existence or not of changes compared to the period prior to the pandemic. |
|  | **WORK EVALUATION** | **Positive_aspects** | General perception of strengths, elements of value and successes in the development of work from the resource throughout the pandemic. Existence or not of changes compared to the period prior to the pandemic |
|  |  | **Obstacles** | Difficulties (internal limitations and external barriers) for the development of work from the services throughout the pandemic. What would have been needed to give a better answer (needs, deficiencies). Existence or not of changes compared to the period prior to the pandemic. |
|  | **RECOMENDATIONS** | **Approach_confinement** | Identification of tools and strategies to carry out prevention, treatment and general approach to IPV during confinement (new cases, aggravation and recidivism). |
|  |  | **Approach_general** | Identification of tools and strategies to carry out prevention, treatment and general approach to IPV. |
|  |  | **Short term_ approach** | Key elements for addressing IPV to be carried out in the coming months. |
|  | **OTHER** | **Other** | Relevant aspects that are not collected in the rest of the codes. |
